# Re-organisation of oesophago-gastric cancer services in England and Wales: a follow-up assessment of progress and remaining challenges

**DOI:** 10.1186/1756-0500-7-24

**Published:** 2014-01-10

**Authors:** Oliver Groene, Georgina Chadwick, Stuart Riley, Richard H Hardwick, Tom Crosby, Kimberley Greenaway, William Allum, David A Cromwell

**Affiliations:** 1 Department of Health Services Research and Policy, Faculty of Public Health and Policy, London School of Hygiene and Tropical Medicine, 15-17 Tavistock Place, London WC1H 9SH, UK; 2 Clinical Effectiveness Unit, Royal College of Surgeons of England, London, UK; 3 Department of Gastroenterology, Northern General Hospital, Sheffield, UK; 4 Cambridge Oesophago-Gastric Centre, Addenbrookes Hospital, Cambridge, UK; 5 Velindre Cancer Centre, Velindre NHS Trust, Cardiff, UK; 6 Clinical Audit Support Unit, The Health and Social Care Information Centre, Leeds, UK; 7 National Cancer Intelligence Network (NCIN), London, UK

## Abstract

**Background:**

This study is an update on an earlier article in 2007 to assess the implementation of the Cancer Plan reform strategy in England and Wales.

**Findings:**

A national online survey to upper gastro-intestinal leads at network and trust level. The questionnaire was designed based on existing clinical practice guidelines and addressed governing principles and operational procedures related to the delivery of cancer care. It was sent in January 2012 to upper gastro-intestinal network and trusts leads at all cancer networks and acute NHS organisations in England and Wales. Responses were received from 100% of Cancer Networks and 91% of NHS organisations. Centralisation of surgery has improved with all but two trusts (5.4%) now meeting the minimum staffing level for oesophago-gastric cancer surgery. This is a substantial improvement since the 2007 survey when 21 trusts (46.7%) did not meet this requirement. The use of formal assessment for nutritional needs has improved, too. In 2007, the involvement of the palliative care team in multi-disciplinary teams was poor. While this has improved, 27 trusts (19.7%) still report that none of the palliative care team members routinely attend the multi-disciplinary team discussion.

**Conclusions:**

The survey demonstrates improved compliance with organisational recommendations since the last assessment in 2007. Centralisation of surgery has improved and is nearly fully compliant with the reform strategy. Areas that require further improvement are nutritional support and inclusion of palliative care in multi-disciplinary team meetings.

## Findings

**Figure 1:**
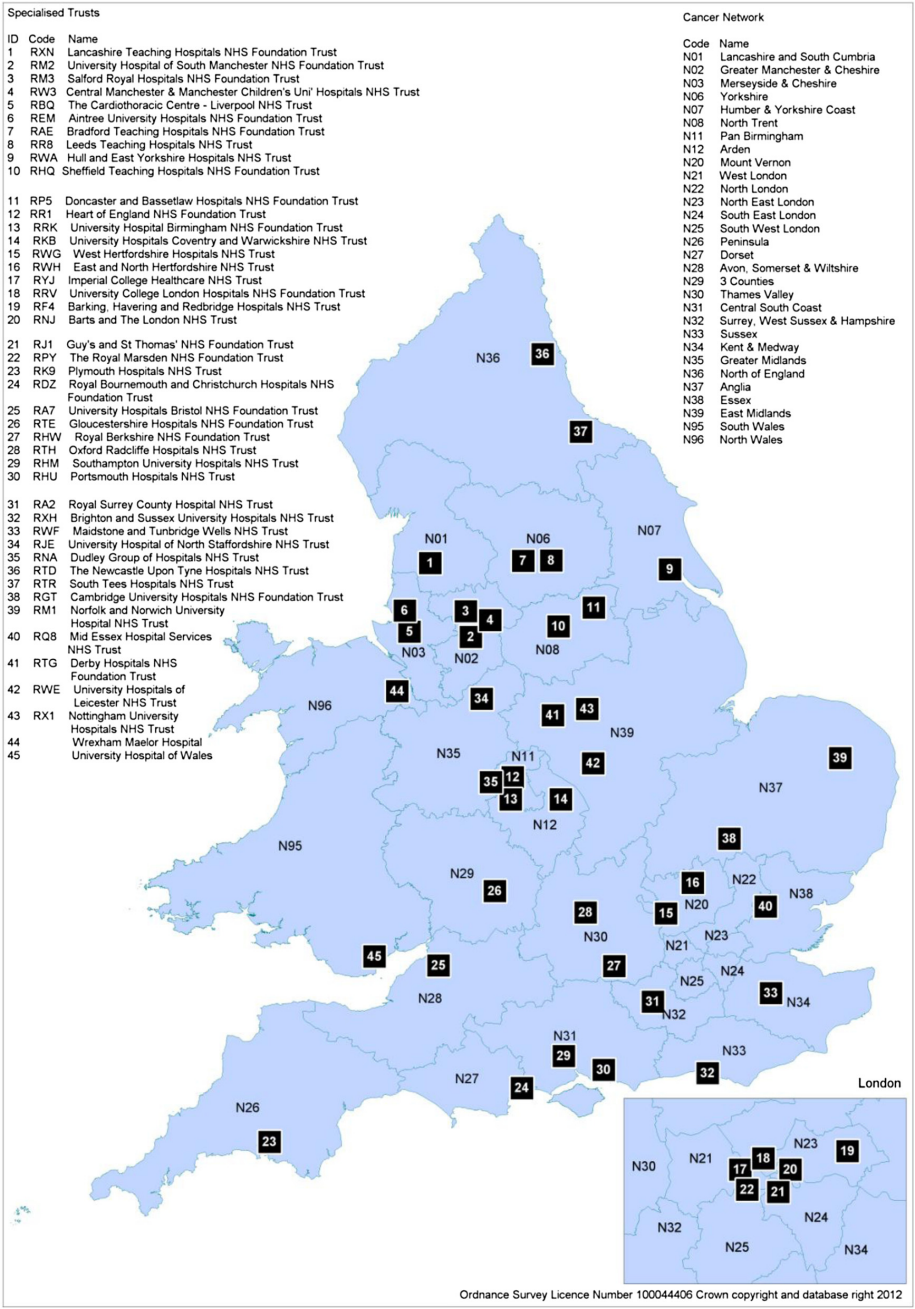
The Cancer Networks in England and Wales that existed on 1 April 2011.

This study is an update on an earlier assessment of the implementation of Cancer Networks for patients diagnosed with oesophago-gastric (O-G) cancer [1]. Cancer Networks were established a decade ago in England and Wales following the Cancer Plan reform strategy with the aim to integrate service provision and improve outcomes [2,3]. Patients diagnosed with oesophageal-gastric cancer (and high-grade dysplasia (HGD), a pre-cancerous alteration of the cells of the oesophagus) require a range of diagnostic, therapeutic and palliative services. The Cancer Plan stipulates that each network should contain one or more specialist cancer centres that provide curative surgical treatment and specialist radiology, oncology and palliative services to all patients living in the area. Diagnostic services and most palliative services continue to be provided by individual NHS organisations (local units) within the network areas. Once a diagnosis is made, all HGD and O-G cancer patients should be discussed in a specialist multi-disciplinary team meeting (MDT) to decide appropriate treatment options. Treatment could include curative surgery (with options for adjuvant/neoadjuvant chemo/radiotherapy) as well as other treatments, which include chemotherapy and or radiotherapy and endoscopic therapies such as stenting, laser ablation, endoscopic mucosal resection and others. Such options should be supported by specialist palliative care teams, and nutritionists. To ensure access to appropriate staging investigations, diagnosis, treatment and palliative care of O-G patients, a clear system of coordination between local units and specialist centres within a network is required (Figure 1).

## Methods

In 2007, an organisational survey of English O-G cancer networks and trust assessed the extent to which the extensive re-organisation of oesophago-gastric cancer services had been completed, and whether the provision of diagnostic, therapeutic and support services was variable across networks and trusts [1]. The survey highlighted that the process of re-organisation was incomplete, in particular with regard to the centralisation of surgery. Moreover, it pointed out that patients were not sufficiently discussed at the specialist MDTs and that access to endoscopic therapies was variable across networks. In addition, dietician support and nutritional assessment of patients and involvement of palliative care teams in the specialist MDTs was insufficient. Recent surveys on the management of Barrett’s Oesophagus, too, highlighted variations in comparison with guidelines and consensus statements [4,5]. Therefore, we conducted a follow-up organisational survey of O-G cancer networks and trusts in order to assess whether variations in management persist and whether progress has been made in the re-organisation and provision of services for O-G cancer in England and Wales since 2007.

Data on the organisation O-G cancer was collected using two separate questionnaires (Additional files 1, 2). The first focused on organisation of services within cancer networks assessing governing principles that inform clinicians, define the scope of care, guide decision-making, and ensure consistency in implementation. Areas of assessment included are referral criteria, organisation of the specialist MDT, access to medical oncology and endoscopic palliative services. The second questionnaire addressed characteristics of services available in individual NHS trusts, focusing on access to specific treatments and relevant professionals.

Questionnaire items were derived from guidelines on management of patients with oesophago-gastric cancer [6-11]. Where possible, we included questions from the previous organisational survey to enable direct comparisons and change of time regarding compliance with recommended standards of care. Additional questions were added to the survey to assess the management of patients with oesophageal high-grade glandular dysplasia (HGD), a precancerous lesion that might developed into oesophageal cancer, as previous studies suggest variability in management and treatment.

A list of networks and all NHS acute trusts involved in the treatment of HGD and oesophago-gastric cancer was prepared from sources at the Health and Social Care Information Centre. Links to the online questionnaires were sent to the Cancer Network O-G cancer lead clinician for the network survey and to trust/unit O-G cancer leads for the NHS trust survey. These links were administered in February 2012 and non-responders were followed up by email and telephone until April 2012. This study was conducted as part of the National Oesophago-Gastric Cancer Audit which has ethical approval and Section 251 approval granted by the Information Governance Board of The Health and Social Care Information Centre (ECC Reference Number: ECC 1–06 © /2011 National Oesophago-gastric cancer audit).

In 2007, 30 Cancer Networks existed, but three Cancer Networks (Leicestershire, Northamptonshire and Rutland, Derby/Burton and Mid Trent) were combined in 2008 to form East Midlands Cancer Network. 154 trusts provided oesophago-gastric cancer services, with 44 trusts being designated specialist centres. In 2012, 28 Cancer Networks existed in England and 151 trusts provided O-G cancer services, of which 39 trusts were designated specialist centres. In Wales, 2 Cancer Networks and 2 specialist trusts existed.

We report the results as the proportion of responding networks and trusts. Where appropriate, differences between specialist and local units were assessed using the Chi-squared test. All p-values are two-sided and those lower than 0.05 were considered to indicate a statistically significant result. STATA 11 was used for all statistical calculations.

## Results

Questionnaires were returned from all Cancer Networks in England (N = 28) and Wales (N = 2). For the NHS trust survey, questionnaires were received from 137 of 151 trusts (90.7%). Completeness of the surveys was very high. Where we report on denominators lower than 30 and 137, respectively, this is mostly due to a) non-applicability of an item or b) 2007 vs 2012 comparisons, where different number of networks and trusts were in place.

### Network level referral criteria and management policy

Of the Cancer Networks, 23 (76.7%) had a specialist surveillance policy for patients with Barrett’s oesophagus, and 26 of the 30 networks (86.7%) had a policy for the management of HGD. In accordance with current guidelines and line with measures of the National Cancer Peer Review Programme, all but one network (96.7%) reported that referral criteria for O-G cancer patients were documented and had a policy to ensure that all oesophageal-gastric cancer patients are referred to and discussed at MDT meetings. In addition, 90% of the networks reported that the policy covered patients with high grade dysplasia as well.

### Implementation of the MDT policy at trust level

All trusts now run MDTs to plan the management of patients and since the last survey a substantially higher number of local units are having combined MDTs with their specialist centres, 63 (70.8%) vs 44 (50.0%) in 2007. Virtually all patients in need of specialist input or eligible for curative resection are now routinely referred to the specialist MDT; however, only 84 trusts (61.3%) referred patients on a best-supportive care pathway. 80 trusts (58.4%) reported discussing all oesophago-gastric cancer patients at the MDT (82.1% at specialist centres vs. 49.0% at local units). Furthermore, 11 (8.0%) trusts do not list private patients at all, and a further 10 (7.3%) reported that private patients were not formally listed. Generally specialist centres were more likely to include private patients in MDT discussions than local units (p = 0.017).

### Organisation and management of patients with High Grade Dysplasia

105 (76.6%) trusts reported that the diagnosis of HGD was confirmed by at least two pathologists with gastrointestinal interest; in line with clinical practice guidelines [8,9]. Of the remaining trusts, 24 (17.6%) reported that diagnosis was confirmed by a pathologist with gastrointestinal interest and 5 (3.6%) stated the diagnosis was based on confirmation by a general pathologist. Responses from 3 trusts (2.2%) were missing. Specialist centres reported a slightly higher compliance of diagnosis in line with clinical practice guidelines, although the difference did not reach statistical significance (p = 0.101).

At present, trusts vary in their mechanisms to ensure referral of HGD patients to an MDT. The most common mechanisms were: referral by clinician (n = 30, 21.9%) or referral by combination of clinician, pathologist and endoscopist (n = 22, 16.1%), referral by combination of clinician and pathologist (n = 16, 11.7%). However, 11 (8.0%) trusts reported having no specific mechanism. The remainder of trusts used other combinations of clinician, pathologist and/or endoscopist referral.

Various endoscopic and surgical options are available for the treatment of HGD and it is recommended that trusts have access to oesophagectomy, endoscopic mucosal resection and at least one of the thermal ablation therapies (argon beam coagulation, multipolar electrocautery, laser therapy, cryotherapy, radiofrequency ablation). The majority of trusts (n = 132, 96.4%) fulfil this recommendation, although there is considerable variability between trusts with regards to the options available to them (Table 1).

**Table 1:** Therapeutic procedures available for patients with high grade dysplasia

**n = 137**	**Available at local trust or access at other hospital in network**	
Procedure	No	Yes
Oesophagectomy	3 (2.2%)	134 (97.8%)
Endoscopic Mucosal Resection	4 (2.9%)	133 (97.1%)
Argon plasma coagulation	21 (15.3%)	116 (84.7%)
Radiofrequency ablation	26 (19.0%)	111 (81.0%)
Photodynamic therapy	37 (27.0%)	100 (73.0%)
Laser therapy	62 (45.3%)	75 (54.7%)
Multipolar electrocautery	81 (59.1%)	56 (40.9%)
Cryotherapy	98 (71.5%)	39 (28.5%)

### Access to curative surgical services in specialist centres

Currently 39 specialist cancer centres provide surgery for oesophageal and gastric resections, responses to the organisational survey were returned by 37 of these. In order to ensure adequate specialist cover 7 days a week including out of hours to manage postoperative complications, the National Cancer Manual recommends that these surgical teams comprise at least three specialist consultant surgeons to manage surgery and postoperative care, although the ideal number would be 4–6 [11]. Only two trusts (5.4%) did not meeting the minimum requirement of having at least three surgeons (Table 2). This is a substantial improvement on the 2007 survey when only 53.3% of centres had three surgeons or more.

**Table 2:** Distribution of surgeons performing oesophago-gastric curative surgery among the specialist O-G cancer centres

**Number of employed or visiting surgeons at the trust**	**2007 (n = 45)**	**2012 (n = 37)**
2	21 (46.7%)	2 (5.4%)
3	14 (31.1%)	13 (35.1%)
4	3 (6.6%)	8 (21.6%)
5	7 (15.6%)	7 (18.9%)
6	0	5 (13.5%)
7+	0	2 (5.4%)

### Access to nutritional support at trust level

Oesophago-gastric cancer patients should have access to dietician advice if needed and should be assessed for nutritional risk using a validated screening tool [8]. Compared to 2007, dietician access has improved for surgical patients in specialist centres. Likewise, nutritional assessment improved with trusts more likely to conduct nutritional assessment and utilise formal screening instruments (Table 3).

**Table 3:** Dietician access and nutritional assessment in specialist centres and local units*

	**2007**		**2012**	
	**Specialist centres n (%)**	**Local units n (%)**	**Specialist centres n (%)**	**Local units n (%)**
***Dietician access***				
Surgical patients	28 (73.7)	NA	33 (84.6)	NA
All other O-G patients	34 (89.5)	75 (85.2)	29 (74.4)	84 (85.7)
Outpatients	32 (84.2)	72 (81.8)	29 (74.4)	74 (75.5)
***Nutritional assessment***				
No formal assessment	9 (23.7)	32 (36.4)	3 (7.7)	15 (15.3)
Dietician assessment	26 (68.4)	43 (48.9)	26 (66.7)	63 (64.3)
Formal screening instrument	3 (7.9)	13 (14.8)	16 (41.0)	38 (38.8)

^*^2007 results based on 38 centres and 88 local units; 2012 results based on 39 centres and 98 local units.

### Access to oncology care and endoscopy procedures in Cancer Networks

As the choice of endoscopic and radiological palliative therapies to treat obstructive oesophageal symptoms depends on individual patient characteristics, availability of a range of treatments is recommended [8]. All Cancer Networks provided access to stent insertion and the majority (85.7%) provided access to argon beam coagulation (Table 4). Brachytherapy and laser ablation could be performed in only about half of the Cancer Networks and is typically performed in specialist centres. Only eight specialist centres (28.6%) offered access to photodynamic therapy, and none of the local units did. Local units in general provided less access to these endoscopic procedures.

**Table 4:** Access to endoscopic procedures in cancer Networks

**n = 28**	**Access to endoscopic procedures in the Cancer Network (either at specialist or local centre)**	
**Procedure**	**No**	**Yes**
Endoscopic stent insertion	0	28 (100%)
Laser ablation	13 (46.4%)	15 (53.6%)
Photodynamic therapy	20 (71.4%)	8 (28.6%)
Argon beam coagulation	4 (14.3%)	24 (85.7%)
Brachytherapy	13 (46.4%)	15 (53.6%)

Eight networks (36.4%) reported that patients had difficulties in accessing oncological therapy within two weeks of the decision to treat. These difficulties applied to both patients receiving both curative and palliative treatment, and did not differ between specialist centres and local units. This is in contrast to 2007, where six networks reported difficulties in accessing oncological radiotherapy, mostly related to access to radiotherapy among specialist centres.

### Provision of palliative care at trust level

In 2007, the involvement of the palliative care team in the MDT was poor, with none of its team members routinely attending the MDT in 10 (26.3%) cancer centres and 26 (29.5%) of local trusts. In 2012, 56 trusts (40.9%) reported that only the specialist nurse attends the MDT while 23 (16.8%) trusts reported that both the consultant and specialist nurse attended the MDT. In addition, 27 trusts (19.7%) stated that none of the palliative care team members routinely attend the MDT meeting. All units now provide access to a palliative care team for patients with oesophago-gastric cancer. All but 4 trusts (2.9%) have implemented an approach to end of life care, with most (n = 129, 94.1%) following the Liverpool Care Pathway.

### Comparing the 2007 and 2012 data

Comparing the data from this survey to the previous survey in 2007 allows assessing how the organisation of services is changing over time. Our analysis suggests that overall policies and operational principles are improving in line with the initial reform plan and recommended standards of care.

The structures and procedures for patients with high-grade glandular oesophageal dysplasia reveal that three quarters of trusts had an agreed policy for management of patients with HGD and followed the recommendation to base diagnosis on confirmation by at least two pathologists with a gastro-intestinal interest [6]. Access to both endocopic and surgical treatment for HGD were generally very good. However, methods of referral to the MDT were variable once the diagnosis was made.

For patients with oesophago-gastric cancer, access to appropriate staging investigations, inclusion in specialist MDTs for curative patients and access to curative services appear good and with little variability across networks. Likewise, dietician support for surgical patients has increased since 2007 even though the use of standardized tools for nutritional assessment is still low. Nevertheless, the proportion of NHS organisations that formally assess nutritional status before treatment has increased significantly.

It appears that less progress has been made in involving palliative care consultants in the care of incurable patients at MDT meetings. This is likely to be due to the national shortage of such clinicians and the practical challenges of them attending multiple weekly MDTs. A fifth of trusts still have no representative from the palliative care team at MDTs. While overall access to palliative endoscopic therapies is good, brachytherapy is poorly available and rarely used. Only 50% of networks have access to this palliative modality and even where it is available it is rarely used. The reasons for this are unclear as brachytherapy is associated with fewer side effects and better palliation than endoluminal stents in patients expected to survive more than three months [12]. Concerning end of life care, the majority of trusts report the implementation of the Liverpool Care Pathway to manage patients in the last days of their life. Further attention should focus on the appropriate planning of palliative community services to reduce admission rates near end of life [13-15].

Since the time of initiating the reorganisation of cancer services, survival of patients undergoing curative surgery has improved from 34% for oesophageal tumours and 40% for gastric tumours to 45% and 50%, respectively [16,17]. Whilst these improvements can not directly be related to the reorganization of services, it is plausible that the concentration of curative surgery brought about improvements in survival in this group of patients. The need for centralisation of cancer services and in particular for cancer surgery has been highlighted by multiple studies showing reduced complications and improved short-term mortality if surgery is performed in a high volume unit [18-20]. Such studies have led to recommendation of minimum volume standards for individual surgeons.

The results of the survey confirm and complement the findings of the National Cancer Peer Review Programme. Peer Review reports steady improvements in the compliance of the 40 Specialist multidisciplinary teams with the peer review measures with the median compliance of 85% (range 52-97%) for all measures in the reviews of 2011/12 [21]. There remain a number of areas where there are centres not meeting the measures and classified as serious concerns by the Peer Review Programme. Although the number of surgeons per centre has increased only 43% are providing a 24/7 on call service. In addition in those centres with more surgeons there are concerns that work is being diluted with fewer procedures being performed by individual surgeons. In some Networks some procedures are still being performed in local units which do not comply with the Department of Health’s Guidance on the Commissioning of Cancer Services. The number of dedicated dieticians has improved but there are still some services where dietician support is deficient. There are three specific measures in the National Cancer Peer Review Programme which are only met in 30% of centres. These are regular review meetings between the specialist centre and the referring diagnostic units to ensure referrals were consistent. Secondly, all those with direct clinical contact with patients should attend an advanced communication skills training programme. Finally at least one clinical core member should have completed training to practice at level 2 for psychological support and this individual should receive a minimum of one hour supervision by a more experienced practitioner. These latter two measures are specifically designed to ensure readily available skilled support for patients and their families. These issues require further investigation.

Overall the response rate for the questionnaire was high, giving weight to the findings. A limitation of the survey is that data is self-reported introducing the risk of ‘social desirability bias’. Since questions addressed facts and responses were provided by key informants at the level of cancer networks and trusts, this bias may be small in this study. Moreover, our findings resonate well with the data collected for the National Cancer Peer Review Programme. Evidently, an assessment of policies and procedures does not allow direct inference to actual compliance with practice recommendations at patient level; however, this will be further assessed by prospective data collection in the National Oesophago-Gastric Cancer Audit [22].

## Concluding remarks

This survey demonstrates improved compliance with organisational recommendations over the last five years, coinciding with the centralisation of services. There is still room to improve supportive services in management of these patients, in particular palliative and nutritional support. Some improvement will need access to greater resources. But simple measures such as ensuring all patients are discussed at MDT and that the palliative care team can attend the MDT may bring about a significant improvement in patient care.

## Competing interests

The authors declare that they have no competing interests.

## Authors’ contributions

OG and DC conceived the study, OG, GC, SR, RH, TC, KG and DC designed the questionnaires, OG conducted the statistical analysis, OG, GC and DC drafted the manuscript, SR, RH, TC, KG and WA revised it for critical intellectual content. All authors read and approved the final manuscript.

## Supplementary Material

Additional file 1 Annex 1: Network Survey. Click here for file

Additional file 2 Annex 2: Trust Survey. Click here for file
