# Supplementary material for: Re-organisation of oesophago-gastric cancer services in England and Wales: a follow-up assessment of progress and remaining challenges
Source: BMC Res Notes. 2014 Jan 10;7:24. doi: 10.1186/1756-0500-7-24 (PMC3896679; doi:10.1186/1756-0500-7-24)
Supplement: Additional file 1 — Annex 1: Network Survey. [file 1756-0500-7-24-S1.doc]

**Annex 1: Network Survey**

This questionnaire forms part of the organisational survey of the National Oesophago-Gastric Cancer Audit (NOGCA). The aim of the Audit is to examine the standard of care received by patients with oesophago-gastric cancer in England, Northern Ireland, Scotland and Wales. The main component of the Audit is a prospective study that will collect data on patients diagnosed between 1 April 2011 and 1 April 2013. The organisational survey aims to examine issues which cannot be covered by the prospective study, such as differences in the availability of various treatment facilities. The organisational survey will involve sending questionnaires to both the network O-G cancer leads and the O-G lead clinicians of individual trusts.

Your contribution to the organisational survey is extremely important. The survey requires a high response rate to ensure its findings are accurate. The results of this survey will be published in the Audit’s first Annual Report in 2012 with our analysis of existing data sources and qualitative study. Together, these should highlight various areas of good performance as well as areas where improvement can be made. If you have any questions relating to the project, please do not hesitate to contact us (see contact details below).

**Data protection statement**

All the information provided on this questionnaire will be treated as confidential. Published reports will only contain aggregated results and will not refer to any individuals or individual organisations.

**Instructions**

Please complete all questions on the questionnaire following the online instructions.

Thank you for your assistance.

Dr Richard Hardwick Dr Stuart Riley Dr Tom Crosby

Lead clinician, AUGIS Lead clinician, BSG Lead clinician, RCR

Contact: oliver.groene@lshtm.ac.uk (methodologist)

kimberley.greenaway@ic.nhs.uk (IC project manager)

**This questionnaire focuses on the organisational policies in place in your network regarding:**

- referral criteria
- organisation of multidisciplinary team
- diagnosis and management of high-grade dysplasia,
- medical oncology and
- endoscopic palliative services

**We understand as ‘policies’ those documented governing principles that inform clinicians, define the scope of care, guide decision-making, and ensure consistency in implementation. In contrast ‘operational procedures’ and ‘guidelines’ intend to put policies into action through a set of measurable actions.**

**There is a maximum of 9 items to be answered. Completing the questionnaire should take only 5 minutes of your time.**

Please enter the name of your network_______________________________

1. **Are referral criteria for oesophageal-gastric cancer patients documented for the whole network?**

- Yes
- No

1. **Do you have a policy to ensure that ALL oesophageal-gastric cancer patients are referred to and discussed at multidisciplinary team meetings (MDTs)?**

- Yes
- No (Filter: go to question 4)

1. **Does that policy explicitly cover high grade dysplasia (HGD) patients?**

- Yes, HGD patients should be discussed at MDT.
- Yes, HGD patients should ***not*** be discussed at MDT.
- No, HGD patients are not covered in the policy.

1. **Do you have a specialist surveillance policy in your network for patients with Barrett’s oesophagus?**

- Yes
- No

1. **Do you have an agreed policy on the management of patients with HGD in your network?**

- Yes
- No

1. **Do any types of patients have difficulty in accessing oncology therapy within 2 weeks of the decision to treat?**

- Yes
- No (**filter**: go the question number 9)

1. **If there are difficulties in accessing oncological therapy within 2 weeks, to which group of patients does that apply?**

- All
- Curative
- Palliative

1. **For which types of therapy do difficulties in access arise?**

**Chemotherapy Radiotherapy**

- - Local Unit □ Local Unit
  - Specialist Unit □ Specialist Unit

1. **What types of endoscopic procedure can be performed at specialist and local units in your network? (tick all that apply)**

a. Endoscopic stent insertion Specialist centres Local units

b. Laser ablation Specialist centres Local units

c. Photodynamic therapy Specialist centres Local units

d. Argon beam coagulation Specialist centres Local units

e. Brachytherapy Specialist centres Local units

**Thank you very much for completing the questionnaire!**
