# Supplementary material for: Re-organisation of oesophago-gastric cancer services in England and Wales: a follow-up assessment of progress and remaining challenges
Source: BMC Res Notes. 2014 Jan 10;7:24. doi: 10.1186/1756-0500-7-24 (PMC3896679; doi:10.1186/1756-0500-7-24)
Supplement: Additional file 2 — Annex 2: Trust Survey. [file 1756-0500-7-24-S2.doc]

**Annex 2: Trust Survey**

This questionnaire forms part of the organisational survey of the National Oesophago-Gastric Cancer Audit (NOGCA). The aim of the Audit is to examine the standard of care received by patients with oesophago-gastric cancer in England, Northern Ireland, Scotland and Wales. The main component of the Audit is a prospective study that will collect data on patients diagnosed between 1 April 2011 and 1 April 2013. The organisational survey aims to examine issues which cannot be covered by the prospective study, such as differences in the availability of various treatment facilities. The organisational survey will involve sending questionnaires to both the network O-G cancer leads and the O-G lead clinicians of individual trusts.

Your contribution to the organisational survey is extremely important. The survey requires a high response rate to ensure its findings are accurate. The results of this survey will be published in the Audit’s first Annual Report in 2012 with our analysis of existing data sources and qualitative study. Together, these should highlight various areas of good performance as well as areas where improvement can be made. If you have any questions relating to the project, please do not hesitate to contact us (see contact details below).

**Data protection statement**

All the information provided on this questionnaire will be treated as confidential. Published reports will only contain aggregated results and will not refer to any individuals or individual organisations.

**Instructions**

Please complete all questions on the questionnaire following the online instructions.

Thank you for your assistance.

Dr Richard Hardwick Dr Stuart Riley Dr Tom Crosby

Lead clinician, AUGIS Lead clinician, BSG Lead clinician, RCR

Contact: oliver.groene@lshtm.ac.uk (methodologist)

kimberley.greenaway@ic.nhs.uk (IC project manager)

**This questionnaire focuses on the operational procedures regarding the organisation of care for oesophageal-gastric cancer patients in your trust, including:**

- organisation of multidisciplinary team
- diagnosis and management of high-grade dysplasia
- informed patient consent
- access to palliative care services, and
- service provision.

**We understand as ‘operational procedures’ those measurable practices that put into action overarching governing principles and policies.**

**There are in total a maximum of 22 items. Completing the questionnaire should take only 10 to 15 minutes of your time.**

Please enter your trust name: ___________________________________

**ORGANISATION OF MULTIDISCIPLINARY TEAM MEETINGS (MDT)**

1. **Does your trust act as a specialist centre for patients with oesophageal-gastric cancer within your network?**

- Yes (**Filter: go to question number 3**)
- No

1. **Does your trust have combined MDT meetings with the specialist centre (e.g. video linked)?**

- Yes
- No

1. **What types of patients are discussed at the specialist centre MDT meetings? Please tick all that apply:**

- Patients needing specialist test available only at the specialist centre
- Those patients thought to be suitable for a curative treatment
- Those patients who need specialist input into their palliation
- Patients on best supportive care pathway
- Patients with High Grade Dysplasia

1. **Are private patients listed for participation in your trust´s MDT meeting?**

- No, not at all **(Filter: go to question 6)**
- No, they are not formally listed but may be discussed in ad hoc manner (**Filter: go to question 6**)
- Yes, they are listed by their clinicians
- Yes, listing is guided by a specific policy on including private patient in the MDT

1. **Is it usually possible to obtain all relevant data for MDT decision making on private patients?**

- Yes
- No

**DIAGNOSIS AND MANAGEMENT OF HIGH GRADE DYSPLASIA (HGD)**

1. **What mechanisms do you have in place to ensure that HGD patients are referred for MDT discussion? (tick all that apply)**

- No specific mechanism
- Pathologists refers directly to MDT
- Investigating clinician refers directly to MDT
- Diagnosing endoscopist refers directly to MDT
- Other MDT referral mechanism

1. **Please indicate your trust´s approach to the diagnosis of HGD:**

- Diagnosis of HGD is always confirmed by a general pathologist.
- Diagnosis of HGD is always confirmed by a pathologist with gastrointestinal interest.
- Diagnosis of HGD is always confirmed by at least two pathologists with gastrointestinal interest.

1. **Is there an agreed management protocol for patients with HGD at your trust?**

- Yes
- No

1. **Are patients diagnosed with HGD at your trust routinely offered endoscopic surveillance?**

- Yes
- No

1. **Please tick if the following procedures to treat patients with HGD are available in your trust, or in case, they are not available, whether you have access to them**

**Available at trust Access at other**

**hospital**

- Oesophagectomy □ □
- Endoscopic Mucosal Resection □ □
- Photodynamic therapy □ □

*Thermal Ablation therapies*

- Argon plasma coagulation □ □
- Multipolar electrocautery □ □
- Laser therapy □ □
- Cryotherapy □ □
- Radiofrequency ablation □ □

**INFORMED PATIENT CONSENT FOR PARTICIPATION IN AUDIT**

1. **Does your trust formally obtain consent for oesophageal-gastric cancer patients participating in any audit (select all that apply)**

- For local audits
- For national audits
- No

1. **Would it be feasible at your trust to obtain informed consent for oesophageal-gastric cancer patients participating in a national audit?**

- Yes
- No

**ACCESS TO PALLIATIVE CARE SERVICES**

1. **Which personnel constitute your palliative care team? (tick all that apply)**

- Consultant in palliative medicine
- Specialist Nurse in palliative care
- Other staff, please specify ___________________________________

1. **Which members of your palliative care team routinely attend the oesophageal-gastric cancer MDT meetings? (tick all that apply)**

- Consultant in palliative medicine
- Specialist Nurse in palliative care
- Other staff, please specify ___________________________________

1. **Do you have an agreed protocol for managing patients whose treatment plan is best supportive care?**

- Yes (please send us your protocol)
- No

1. **Have you implemented at your trust any of the following approaches to care for people in the last days of life? (tick all that apply)**

- Gold standards framework
- Liverpool care pathway
- NICE guidance on supportive and palliative care for adults with cancer
- Preferred priorities for care

**NUTRITIONAL SUPPORT**

1. **At your trust, which patients with O-G cancer have access to specialist nutritional advice from a dietician? (tick all that apply)**

- Surgical inpatients only
- All other O-G cancer inpatients
- O-G cancer patients when seen as an outpatient

1. **Typically, at your trust, how is the nutritional status of O-G cancer patients formally assessed prior to treatment (such as surgery or chemotherapy)?**

- There is no formal assessment prior to treatment
- Dietician assessment
- Assessment using a standard screening instrument, eg. Nutritional Risk Index (NRI)
- Using a locally-developed screening instrument
- Other method, please specify ___________________________________

**SERVICE PROVISION**

1. **How many specialist nurses for O-G cancer are employed at your hospital (excluding nurse endoscopists)?**

Full-time _______________

Part-time_______________ (include nurses shared with other specialties)

Part-time_______________ (nurses who come from another hospital)

1. **Does the trust undertake curative surgery (resection) for patients with OG cancer? Tick all that apply.**

- Oesophageal resection
- Gastric resection
- No surgery performed

1. **Does the trust have visiting surgeons from local cancer units to undertake curative surgery for OG cancer? Tick all that apply.**

- For gastric resections
- For oesophageal resections
- No

1. **How many surgeons perform resection procedures at this trust?**

Employed at the trust:

Upper GI surgeons _______

Thoracic surgeons _______

Visiting surgeons _______

**Thank you very much for completing the questionnaire.**
